# Quadricuspid pulmonary valve with pulmonary hypertension: a case report

**DOI:** 10.1093/ehjcr/ytae408

**Published:** 2024-11-27

**Authors:** Wenting Li, Yang Zhang, Xiaopei Cui, Qiushang Ji

**Affiliations:** Department of Radiology, Qilu Hospital of Shandong University, 107 Wenhua Xilu, Jinan, Shandong 250012, P.R. China; Department of Radiology, Qilu Hospital of Shandong University, 107 Wenhua Xilu, Jinan, Shandong 250012, P.R. China; Department of Geriatric Medicine, Qilu Hospital of Shandong University, 107 Wenhua Xilu, Jinan, Shandong 250012, P.R. China; Department of Cardiology, Qilu Hospital of Shandong University, 107 Wenhua Xilu, Jinan, Shandong 250012, P.R. China

**Keywords:** Pulmonary valve, Adult congenital heart disease, Diagnostic imaging, Case report

## Abstract

**Background:**

The quadricuspid pulmonary valve (QPV) is a relatively rare heart congenital anomaly. It is usually asymptomatic and incidentally detected.

**Case summary:**

A 52-year-old woman presented with paroxysmal palpitations and chest tightness after exertion. After a series of examinations, we finally diagnosed her with QPV and pulmonary hypertension. The symptoms have improved significantly with medications, and subsequently, the patient was discharged.

**Discussion:**

This case demonstrated the crucial role of multimodality imaging in evaluating the non-invasive depiction of pulmonary valve disease.

**Learning points**

- The quadricuspid pulmonary valve (QPV) is relatively rare. The QPV is usually asymptomatic and incidentally detected, but sometimes, it can be associated with other cardiac abnormalities.
- This is the first case of a QPV combined with unclassified pulmonary hypertension, although QPV seemingly appeared as a bystander. Pulmonary hypertension is what we need to pay close attention to.
- This case also demonstrated the crucial role of multimodality imaging in the diagnosis and visualization of morphological valvular features and associated insufficiency in QPV in living patients.

## Introduction

The normal semilunar valve, including the aortic and pulmonary valve, typically consists of three leaflets. Abnormalities in the number of leaflets are observed mainly in the aortic valve, such as the unicuspid or bicuspid aortic valve. The quadricuspid pulmonary valve (QPV) is a relatively rare heart congenital anomaly. The QPV tends to be clinically asymptomatic and is generally identified in post-mortem autopsy. The prevalence of QPV in post-mortem specimens has been reported to range from 1 in 400 to 1 in 1000.^1^

## Summary figure

**Figure:**
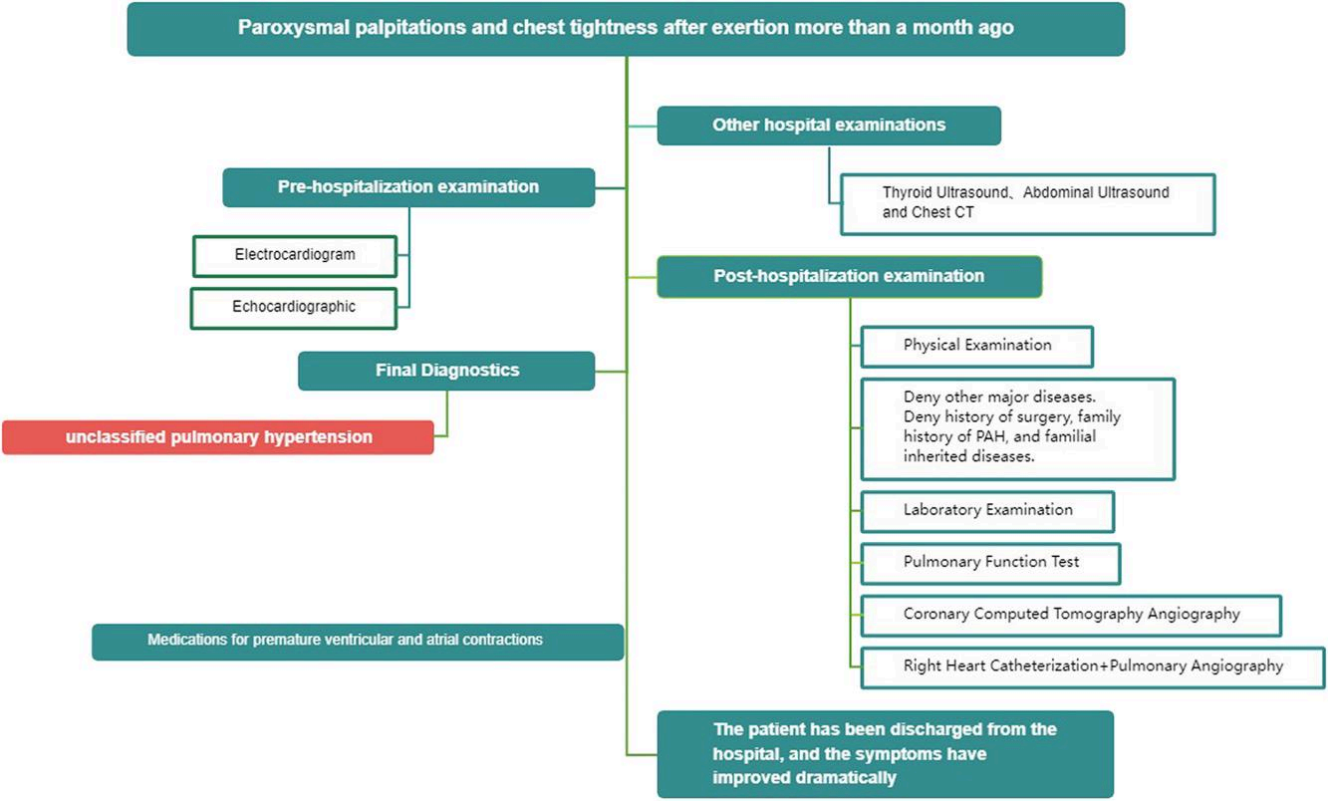


The QPV is predominantly isolated but sometimes can be associated with other cardiac abnormalities.^1–3^ The most frequently encountered associated abnormalities include patent ductus arteriosus, atrial septal defects, ventricular septal defects, and aortic valve abnormalities. We report a case of QPV with pulmonary hypertension, which was identified on the post-admission incidentally.

## Case presentation

**Figure 1:**
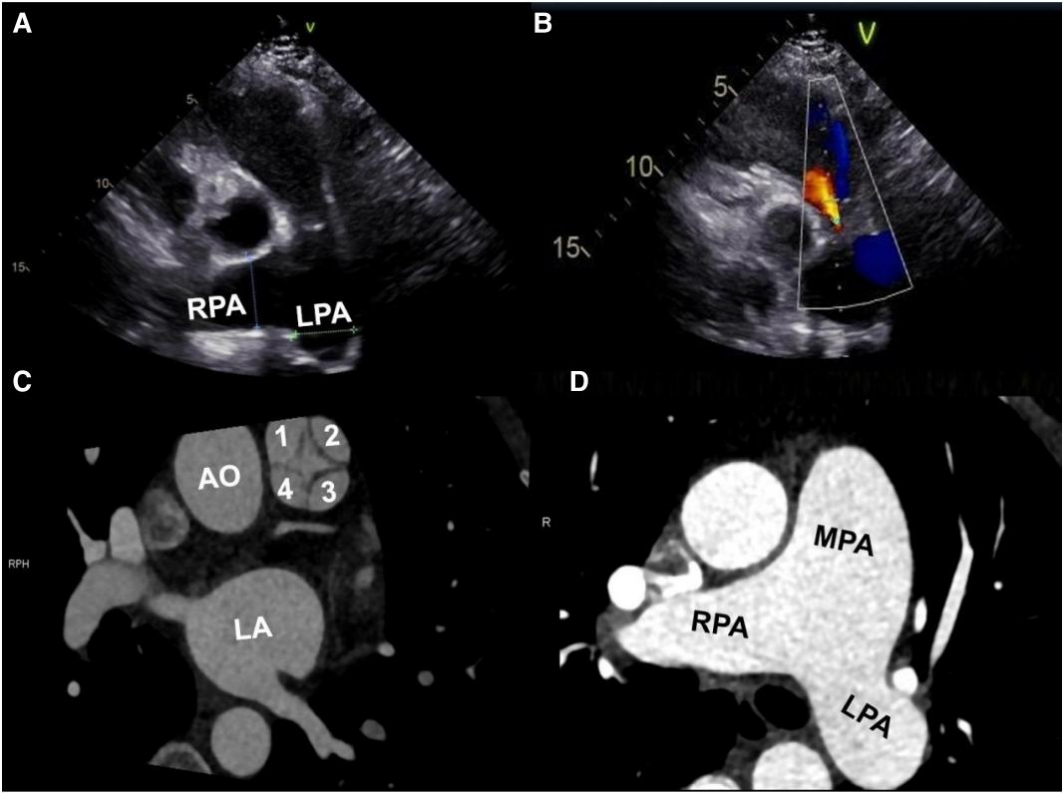
Images in a 52-year-old woman. (*A*) The transthoracic echocardiographic image revealed dilation of the pulmonary arteries of the left (25 mm) and right (28 mm). (*B*) Colour Doppler imaging showed mild pulmonary valve regurgitation and systolic flow acceleration time in the right ventricular outflow tract (63 ms). (*C* and *D*) The computed tomography angiography scan demonstrated a quadricuspid pulmonary valve (leaflets are numbered 1, 2, 3, and 4) with one smaller cusp (leaflet number 4) and a dilated main pulmonary artery (33 mm). AO, aorta; LA, left atrium; MPA, main pulmonary artery; RPA, right pulmonary artery; LPA, left pulmonary artery.

A 52-year-old woman presented with paroxysmal palpitations and chest tightness after exertion more than a month ago, especially at night, and could be improved after rest. The patient did not have a specific past medical history or major illnesses. The physical examination revealed vital signs within normal limits, including blood pressure (125/76 mmHg) and heart rate (67 b.p.m.). Cardiac examination was unremarkable, with normal heart sounds and no murmurs or rubs. Holter monitor depicted normal sinus rhythm overall, with premature ventricular contractions and premature atrial contractions. Transthoracic echocardiography findings included the following: left and right pulmonary artery dilatation (*Figure 1A*), mild pulmonary valve regurgitation (*Figure 1B*), and mild pulmonary hypertension (see Supplementary material online, *Data S1*). The right heart was enlarged with normal right ventricular function. The normal left ventricular size and systolic function, with an ejection fraction of 60%, both the mitral and tricuspid valves, showed normal structure and function.

Moreover, pulmonary function tests showed normal pulmonary ventilation function. A coronary computed tomography angiography (CTA) excluded coronary artery disease. Incidentally, it revealed a QPV with three similar-sized cusps and one small (*Figure 1C*). At the same time, we found a dilated pulmonary trunk (about 33 mm; *Figure 1D*) and right ventricular hypertrophy. Subsequent right cardiac catheterization suggested pulmonary hypertension (see Supplementary material online, *Data S2*). Pulmonary angiography showed that there was no significant filling defect in the pulmonary artery and its branches.

Other hospital examinations including thyroid ultrasound, abdominal ultrasound, and chest computed tomography (CT) suggested no abnormality.

Finally, we ruled out other types of pulmonary hypertension and classified this as QPV with ‘not classified’ pulmonary hypertension.

### Follow-up

Currently, the patient has been discharged from the hospital, and the symptoms have improved dramatically with medications for premature ventricular and atrial contractions, such as metoprolol succinate sustained-release tablets. We did not give this patient targeted anti-pulmonary hypertension therapy because her pulmonary vascular resistance was not higher. We plan to follow up with the patient for the long term.

## Discussion

In this case, the QPV was discovered incidentally by coronary CTA. According to the Hurwitz and Roberts^4^ classification, QPV is categorized into seven types, the most common of which consists of three similar cusps and one smaller cusp (Type B), as in the present case.

While transthoracic echocardiography is not the optimal diagnostic tool for detecting QPV due to its anatomical location relative to the thoracic wall, it can provide valuable information regarding the function of the valve. Cardiac CT allows an adequate diagnosis and visualization of morphological features of the valve.

Furthermore, it is worth mentioning that this patient had combined pulmonary hypertension (not classified). In previous cases^5,6^ with QPV with pulmonary hypertension, these findings were considered as resulting from left heart failure or advanced cardiac disease (Group 2).^7^ To our knowledge, this is the first case of QPV combined with not classified pulmonary hypertension. The clinical symptoms of the patient improved dramatically with medications for premature ventricular and atrial contractions, seemingly suggesting that the QPV was an innocent bystander. Our goal in presenting this case is to offer fresh perspectives that contribute to the existing knowledge in the literature.

## Conclusion

The QPV is relatively rare. Echocardiography and cardiac CT can provide useful anatomic and functional information to support diagnosis and subsequent clinical management.

## Supplementary Material

ytae408_Supplementary_Data

## Data Availability

No new data were generated or analysed in support of this research.

## Lead author biography

Dr Wenting Li is a post-graduate student in the Department of Radiology at Qilu Hospital of Shandong University, where she received her training. The main research fields include valvular heart disease and cardiac imaging.

## Notes

**Consent:** In line with the COPE guidelines, written informed consent was provided by the patient for publication of this case report and accompanying images.

**Ethics approval:** This case report has been approved by the Qilu Hospital of Shandong University Ethics Committee.

**Funding:** This study has received funding from the Major Science and Technology Innovation Projects of Shandong Province (No. 2021SFGC0104).
