# Supplementary material for: Quadricuspid pulmonary valve with pulmonary hypertension: a case report
Source: Eur Heart J Case Rep. 2024 Nov 27;8(11):ytae408. doi: 10.1093/ehjcr/ytae408 (PMC11600956; doi:10.1093/ehjcr/ytae408)
Supplement: ytae408_Supplementary_Data [file ytae408_supplementary_data.docx]

**[Supplementary data](https://oup.silverchair-cdn.com/oup/backfile/Content_public/Journal/ehjcimaging/19/12/10.1093_ehjci_jey113/1/jey113_s1.mp4?Expires=1716689348&Signature=CDqkOgAA6hQEMzXUcjmjJfIyfpyxvM8T3YI7l8ObDpJdFOj6rCfxyFr9Fst2Uu6J8sCrg86F3DdoFyKoQ82Ut~PL7ML4W8YAZBa2RbdqXdz0fitYz4Bx5UUJXT51Wo0dVL3O-A7~AmQd0ldfWdc82mx7Sg2z74lXKlRG5q4FOmd5O1d77fHdMz6NPxcSAlyQhBfpdDEchqiA3j8yvEt7Ev1aWel5DPqfNlINtB7VR6PJevEUYF54zaTnjKnK7bqm0zX3eF5PCrnMmFLk-40bhVFSsGDSOKbLECkUtbJBivqeT2QJtPLHVjbnU3J18NW24WH3GWqkEv5KCX3xikwBjw__&Key-Pair-Id=APKAIE5G5CRDK6RD3PGA)**

**S1**

The echocardiographic parameters that support the diagnosis of pulmonary hypertension in this patient are as follows: (1) Direct sign: **Pulmonary artery systolic pressure(PASP) of about 44 mmHg estimated by the tricuspid regurgitation pressure gradient method. Pulmonary artery mean pressure, estimated by the pulmonary regurgitation pressure gradient method, is approximately 29 mmHg. (2) Indirect sign: The right ventricular outflow tract acceleration time was 63ms(＜105ms).** The area of the right atrium was 19 cm²(＞18cm²).

**S2**

Right cardiac catheterization showed: Pulmonary arterial pressure(40/13/22 mmHg), Pulmonary arterial wedge pressure(15/7/9 mmHg), Pulmonary vascular resistance(1.97 WUs) ,right atrial pressure (7/-1/2 mmHg), right ventricular pressure (40/-2/12 mmHg), Cardiac output(6.6 L/min), and Cardiac index(3.82 L/min/m^2^).
